# Ophthalmic Research Priorities and Practices in Nigeria: An Assessment of the Views of Nigerian Ophthalmologists

**DOI:** 10.4103/0974-9233.80707

**Published:** 2011

**Authors:** Abdulraheem O. Mahmoud, Abdulkabir A. Ayanniyi, Abdu Lawal, Charles O. Omolase, Yinka Ologunsua, Elsie Samaila

**Affiliations:** Department of Ophthalmology, University of Ilorin Teaching Hospital, Ilorin, Nigeria; 1 Department of Ophthalmology, University of Abuja, Abuja, Nigeria; 2 Department of Ophthalmology, Aminu Kano Teaching Hospital, Kano, Nigeria; 3 Department of Ophthalmology, Federal Medical Center, Owo, Nigeria; 4 Eye Unit, St Mary’s Catholic Hospital, Ago Iwoye, Nigeria; 5 Department of Ophthalmology, Ahmadu Bello University Teaching Hospital, Shika-Zaria, Nigeria

**Keywords:** Ophthalmic Research, Ophthalmic Research Priorities, Health Research Utilization

## Abstract

**Purpose:**

To study the views of ophthalmologists on research priorities and outcomes in Nigeria.

**Materials and Methods:**

A structured questionnaire was distributed to 120 ophthalmologists and ophthalmic residents who were attending an annual congress in Nigeria. The participants’ background information, relative research priorities, frequency of publications, research types, publication media, challenges faced in publishing and impact on health practice or policy were collected.

**Results:**

Eighty-nine (74.2%) of the 120 questionnaires were returned. Childhood blindness was given the highest priority for ophthalmic research by 42.9% of the respondents, and genetic studies had the least priority (19.8%). About two-thirds of the respondents had either never been involved or only involved occasionally in any type of ophthalmic research. Clinical trials (13.1%) and basic science studies (12%) were the least-performed types of research. About 51% of the respondents indicated that they had never published in journals nor did so “occasionally”; only 9% quarterly and 43% published less than once a year. They also indicated that their research very rarely resulted in change of clinical practice or health policy (20%).

**Conclusions:**

Research works conducted by respondents were largely simple low-budget ones that rarely had significant impacts and outcomes, including publication. There is a need to retrain and emphasize the importance of research during undergraduate and postgraduate medical education. Adequate resources and research infrastructure should be provided for ophthalmic research in Nigeria.

## INTRODUCTION

Nigeria has a population of 140^1^ million and a significant health burden of blindness due to various diseases.^2^ The prevalence of blindness (<20/400 in the better eye) is 4.2% and severe visual impairment (presenting vision <20/200 to 20/400) is 1.5% in the general population that is 40 years or older.^2^ In Nigeria, blindness is associated with increasing age, being female, poor literacy and residence in the North.^2^ It is estimated that 4.25 million adults 40 years or older have moderate to severe visual impairment or blindness (<20/63 in the better eye).^2^

There is one ophthalmologist for every 350,000 people in Nigeria. The shortage of ophthalmologists is further compounded by the inadequate geographic distribution, as most practice in tertiary health facilities in large cities. Furthermore, their expertise is inappropriately utilized as they attend mainly to relatively minor ophthalmic ailments that could be delegated to allied ophthalmic personnel.^3^

The persistent crippling burden of disease in the African region as a whole can be attributed to a number of causes, including weak national and district health systems, a crisis in human resources in the health sector that has been exacerbated by an internal and external brain drain. Other factors include the lack of access to health services in 47% of the population, lack of access to essential drugs to approximately 50% of those in need,^4^ 59% of pregnant women deliver babies without the assistance of skilled health personnel,^5^ 64% of the population lack sustainable access to improved sanitation facilities and 42% lack sustained access to an improved water source,^6^ out-of-pocket expenditures constituting 51–100% of the private health expenditure,^5^ 38.2% of the population in sub-Saharan Africa living below the international income poverty line of US$1 per day,^7^ low investment in health development and poor governance.^8^ These challenges are compounded by weak national health research systems, which hinder the generation of new data and knowledge for diagnosing and providing solutions; monitoring of health system performance; development and implementation of new technologies and health products for tackling priority diseases and health conditions; and innovation in national health care access and implementing cost-effective, preventive, curative and rehabilitation interventions.^9^

The volume of peer review research publications from Nigeria is largely relative to most African countries.^10^ However, there is a palpable dearth of published works on the general perceptions of the individual health researchers in Nigeria, particularly ophthalmologists, on the various challenges that they face in conducting research. Such studies on perceptions of researchers in both developing countries^11^,^12^ and developed countries^13^–^15^ have actively shaped health care research. Studies on ophthalmic research activities have also been conducted in the Far East^16^ and in the Nordic countries^17^ to quantify the research efforts by ophthalmologist and scientists. In this study, we study the opinion of ophthalmologists on research priorities, practices and outcomes in Nigeria, and make suggestions to improve the ophthalmic research activity in Nigeria.

## MATERIALS AND METHODS

This study was part of a wider study on various facets of research undertaken by ophthalmologists in Nigeria. This portion of the study concentrated on collating the views of ophthalmologists on research priorities, practices and outcomes in Nigeria. Research ethics board approval for the study was obtained from the Aminu Kano Teaching Hospital, Kano, Nigeria.

Questionnaires for this study were distributed and collated at the 34^th^ Annual Congress and Scientific Conference of the Ophthalmological Society of Nigeria, held in Lagos, Nigeria, between the 14^th^ and 17^th^ September, 2009. Copies of the study questionnaire were distributed to the 120 ophthalmologists and ophthalmic residents who attended the conference. The self-administered, anonymous questionnaire was distributed after full confidentiality of the collected data was ensured to all the study participants and the assurance that the results of this study would not be presented either for an individual study participant or for a hospital. Pre-testing was performed prior to initiating the study by administering the questionnaire to a sample of ophthalmologists to assess comprehension and feasibility. The results of pre-testing indicated the need to broaden the latitude of the response options to 4 (0–3) rather than limit it to fewer options, such as categorical yes or no options.

In all, 14 questions were included in the study questionnaire. The first four questions gathered information on the study participants’ demographic and professional background. The remaining 10 questions queried relative research priorities on the diseases and type of ophthalmic research, frequency and type of research, publication preference, challenges faced in getting the work published and the impact of the research in changing health practices or health policy. For each of the 10 questions that were all independent of each other, respondents assessed a subset of four to five sub-sections, on a scale of 0-3, with 0 representing none/never/lowest/least and 3 representing most/highest/greatest/always/strongest, depending on the specific context of the question posed, with the respondents’ choosing appropriate responses. Data were analyzed with SPSS version 15.0 (SPSS Inc, Chicago, IL, USA) to generate descriptive statistics such as frequencies, percentages and proportions. The Chi-square test was used to test significant differences. A *P*-value of less than 0.05 was statistically significant.

## RESULTS

Eighty-nine of the 120 (74.2%) questionnaires that were distributed were returned for data analysis. The study participants were constantly reminded throughout the duration of the 4-day conference to fill and submit their questionnaires in order to ensure a high response rate.

### Background data

The mean age of the respondents was 41.65 ± 7.24 years (range, 24–63 years). Forty-three of the 87 (49%) respondents who indicated their gender were males. Of the 86 who stated their professional status, 51 (59.3%) were fellows/consultant ophthalmologists with postgraduate fellowship training, 12 (13.9%) were senior ophthalmic residents, 18 (20.9%) were junior ophthalmic residents and five (5.8%) were diplomates, i.e. mid-level ophthalmologists with intermediate/diploma certificates from national or international ophthalmology postgraduate training colleges. Of the 53 respondents who indicated their years of their ophthalmic practice experience, 25 (47.2%) practiced for less than 5 years, eight (15.1%) practised for 5-9 years, 11 (20.8%) practiced for 10–15 years and 17.0% practiced for more than 15 years.

### Disease priority for research

Childhood blindness (42.9%) and refractive errors (38.8%) were considered the highest priority of ophthalmic research among respondents. Genetic/familial ophthalmic conditions were given the least priority rating for research, with only 19.8% of the respondents considering genetic/familial research the highest priority. Table 1 provides the priority and topics of ophthalmic research among respondents.

**Table 1:** Rating of the priority for some diseases for health research by Nigerian ophthalmologists

Disease	Number of respondents	None (%)	High (%)	Higher (%)	Highest (%)	Total (%)
Age-related macular disease	86	6 (7.0)	26 (30.2)	35 (40.7)	19 (22.1)	86 (100.0)
Childhood blindness	84	6 (7.1)	15 (17.9)	27 (32.1)	36 (42.9)	84 (100.0)
Refractive errors	85	3 (3.5)	11 (12.9)	38 (44.7)	33 (38.8)	85 (100.0)
Ocular tumors	85	5 (5.9)	32 (37.6)	31 (36.5)	17 (20.0)	85 (100.0)
Genetic/familial eye disease	86	16 (18.6)	28 (32.6)	25 (29.1)	17 (19.8)	86 (100)

### Types and class of health research practices

**Figure 1:**
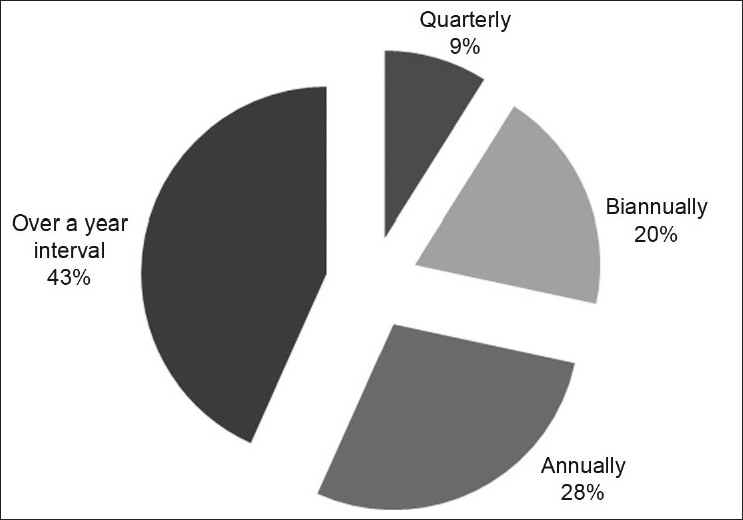
Frequency of publications among Nigerian ophthalmologists (*n* = 67)

Six respondents out of the 67 (9.0%) published quarterly, 13 (19.4%) biannually, 19 (28.4%) annually and 29 (43.3%) less than once a year [Figure 1]. The age (*P* = 0.186), sex (*P* = 0.096), cadre (*P* = 0.227) and duration of postgraduate fellowship training (*P* = 0.452) were not significantly associated with the frequency of publication.

At least 50% of the respondents had either never been involved in or only involved occasionally/rarely involved in general health research. Clinical trials were never or rarely conducted by 66.6% of the respondents. Educational research for medical qualifications (e.g., for studies in undergraduate and postgraduate medical degrees) was conducted by at least 52.3% of the respondents. Table 2 presents the frequency of involvement in the different types of research.

**Figure 2:**
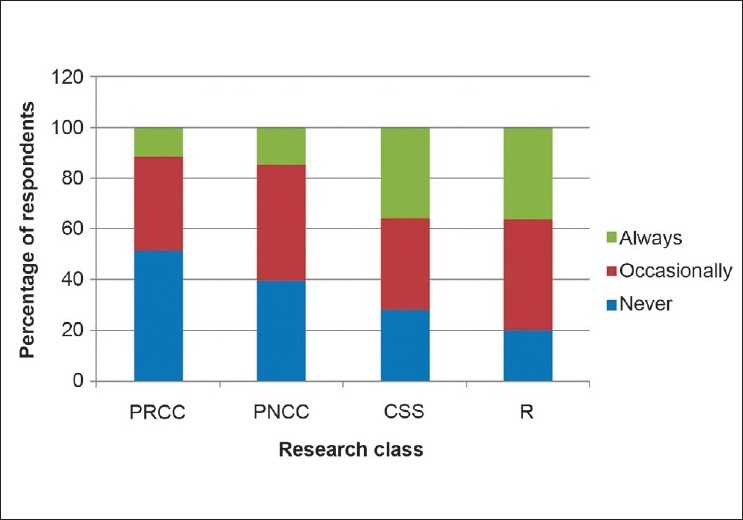
Rating of the frequency of involvement in different classes of ophthalmic research by Nigerian ophthalmologists (*n* = 80)

Between 78.2% and 36.3% of the respondents for each category of research had only occasionally or never being involved in ophthalmic research. Fifty-one percent and 39.5% of the respondents had never been involved in a prospective, case-controlled randomized study or a prospective, non-randomized study, respectively. Retrospective studies and cross-sectional studies were being conducted “always” by at least 36.3% and 35.9% of the respondents in each category, respectively.Figure 2 gives the details of the involvement of the respondents in the different classes of research.

**Table 2:** Frequency of involvement in different types of health research by Nigerian ophthalmologists

Research type	Number of respondents	Never (%)	Occasionally (%)	Sometimes (%)	Always (%)	Total (%)
Clinical trials	84	49 (58.3)	7 (8.3)	17 (20.2)	11 (13.1)	84 (100.0)
Basic/applied science	83	27 (32.5)	30 (36.1)	16 (19.3)	10 (12.0)	83 (100.0)
Health system research	80	19 (23.8)	28 (35.0)	19 (23.8)	14 (17.5)	80 (100.0)
Educational*	84	20 (23.8)	20 (23.8)	27 (32.1)	17 (20.2)	84 (100.0)

* E.g. various aspects of undergraduate and postgraduate medical education

### Medium of publication

**Figure 3:**
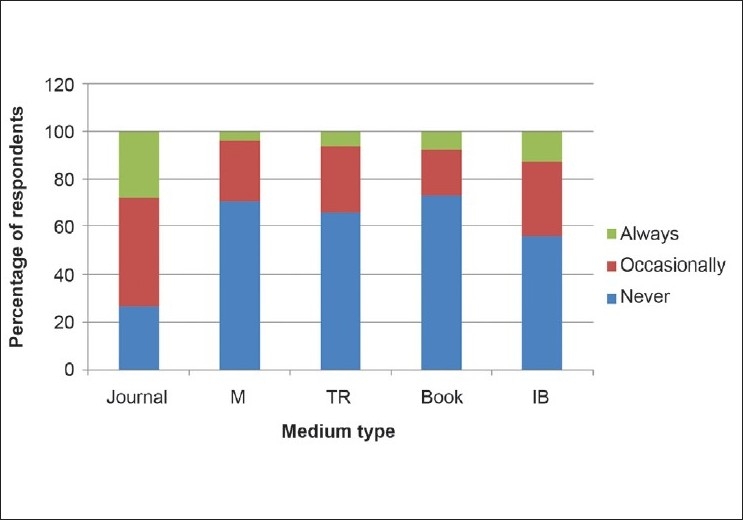
Rating of the frequency of publications in different types of media by Nigerian ophthalmologists (*n* = 79)

Journals appeared to be the most favored media to publish research findings, with 49.4% indicating that they published “always” or “sometimes” in journals. Publications in monographs, books and technical reports were least preferred [Figure 3]. The age (*P* = 0.817), sex (*P* = 0.271), cadre (*P* = 0.873) and duration of postgraduate fellowship training (*P* = 0.245) were not significantly associated to internet-based publications such as open-access journals.

The majority of the respondents had either never published or occasionally published in all the different classes of publication media itemized in the questionnaire. The Nigerian-based Pub Med-indexed journals (22%) and local institutional/departmental documents (20.3%) were the preferred publication medium. Table 3 presents the details regarding preference of publication media.

**Table 3:** Rating of the frequency of publications in different classes of media by Nigerian ophthalmologists

Class of medium	Number of respondents	Never (%)	Occasionally (%)	Sometimes (%)	Always (%)	Total (%)
Foreign-based and indexed*	81	48 (59.3)	17 (21.0)	7 (8.6)	9 (11.1)	81 (100.0)
Foreign-based and not indexed*	77	51 (66.2)	17 (22.1)	6 (7.8)	3 (3.9)	77 (100.0)
Nigerian-based and indexed*	82	36 (43.9)	17 (20.7)	11 (13.4)	18 (22.0)	82 (100.0)
Nigerian-based and not indexed*	78	40 (51.3)	17 (21.8)	13 (6.7)	8 (10.3)	78 (100.0)
Local institution/departmental	74	26 (35.1)	25 (33.8)	8 (10.8)	15 (20.3)	74 (100.0)

* Publication media indexed in Pub Med

The study respondents indicated that they published their research as “very challenging” because of the lengthy publication processes (28.4%), difficulty in identifying suitable medium (21.3%), publication fees (20.3%) and publisher/editorial bias (20.5%) [Table 4]. On the issue of whether the respondents had experienced the issue of a serious conflict of interest being raised when trying to publish their research, 76 of the 79 respondents who answered (96.2%) this question had never had such an experience, while three (3.8%) had faced such a scenario.

**Table 4:** Challenges faced by Nigerian ophthalmologists in getting their research published

Challenge	Number of respondents	Not challenging (%)	Mildly challenging (%)	Somewhat challenging (%)	Very challenging (%)	Total (%)
Identifying suitable medium	75	13 (17.3)	15 (20.0)	31 (41.3)	16 (21.3)	75 (100.0)
Lengthy publication processes	74	6 (8.1)	19 (25.7)	28 (37.8)	21 (28.4)	74 (100.0)
Publication fees	74	15 (20.3)	20 (27.0)	24 (32.4)	15 (20.3)	74 (100.0)
Editorial/publishers bias	73	18 (24.7)	21 (28.8)	19 (26.0)	15 (20.5)	73 (100.0)

### Inventions and policy change outcomes

Three (7.8%) of the 79 respondents that answered indicated that they had a registered patent for inventions made, such as new ideas, procedures, medicaments and appliances, etc., while the majority [76 (92.2%)] had no patents.

**Figure 4:**
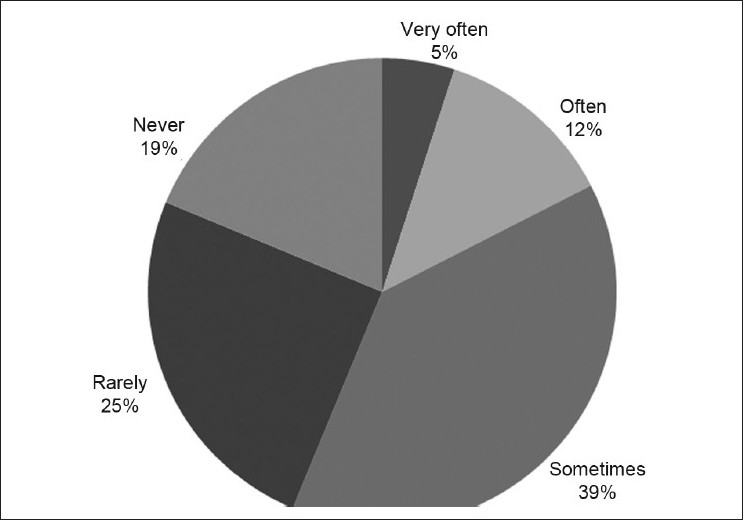
Distribution of respondents by how their research resulted in changes in health policy or practice (*n* = 80)

Up to 25% (20/80) of the respondents rarely and 19% (15/80) never had their research findings result in a change in health policy or practice [Figure 4].

## DISCUSSION

A previous study of ophthalmic research activities in Asia inferred that the significant burden of blinding eye diseases results in a disproportionately greater importance on clinical service and teaching, resulting in research being given a low priority in the hospital and departmental setting.^16^ However, once there is successful implementation of the basic health infrastructure and services, the Asian countries are now channelling resources to ophthalmic research, which is resulting in numerous high-quality publications in leading ophthalmic journals.^16^ This outcome is instructive to Nigeria, which also has a significant health burden of blindness and is still developing the provision of basic eye health.^3^ Consequently, ophthalmic research at present appears to receive low priority among clinicians and scientists in Nigeria.

In our study, the finding that the demographic (age and sex) and professional (cadre and years of postgraduate fellowship training) backgrounds of the respondents generally did not have any statistically significant association with responses needs to be examined further. There was no significant association even on issues such as the frequency of publication, where possibly males and younger respondents would be expected to publish more frequently; and younger respondents possibly being more internet savvy to publish on an internet-based medium. The lack of statistical significance is likely due to the small number of respondents who were publishing.

Childhood blindness and refractive errors were given the high priority for research in this study. Childhood blindness and refractive errors rank among the five leading causes of blindness worldwide, for which effective and cost-efficient interventions are available.^18^ Sommer^19^ pointed out the opportunity to conduct unique clinical research in resource-challenged sub-Saharan countries such as Nigeria due to the fact that some clinical conditions are unique to poor populations or occur at far higher rates than in populations elsewhere (trachoma, onchocerciasis, xerophthalmia, loa-loa, agricultural injuries). Other facets identified by Sommer^19^ included the larger subject pool that can make for more definitive diagnostic criteria, populations in which a disease is more prevalent are more efficient for conducting treatment trials and resource-poor populations provide the only relevant opportunity for studying and testing simplified interventions appropriate to low-resource settings.

The respondents in our study generally indicated a poor enthusiasm for any type and class of research, and the few research works that were being conducted were largely simple low-budget retrospective studies, cross-sectional ones and educational studies. This is most probably a reflection of poor access to sources of research funding by the respondents, an aspect analyzed in a different segment of our larger study. Additionally, the lack of familiarity with complex research such as prospective randomized trials in 51.3% of the respondents is another factor in the willingness to conduct research.

More disconcerting is the poor involvement of the respondents with health system research, which is not usually technologically nor capital intensive and is crucial to help achieve the goals of Vision 2020: The Right to Sight and the health-related Millennium Development Goals.^20^ Finding and retaining adequate financial and human resources to conduct health research is a major problem, especially in low- and middle-income countries, where the need is often greatest.^19^ Innovative research training modules utilized in Ghana (a West African country similar to Nigeria) that enabled interested participants to design and undertake a novel course that developed individual and institutional research capacity that met international standards^21^ should be made readily available to other developing countries.

Publications may be viewed more as a measure of productivity rather than as research impact^22^–^24^ or outcome; the problems for African researchers (including ophthalmologists) are compounded by the fact that there are only a few medical journals published in Africa, some of which are published irregularly and are “probably” of low quality.^25^

The outcome that research findings made little or no impact on either clinical practice or health policy in this study and another study in Nigeria^26^ could be due to poor topics lacking relevance and poor dissemination of the research outcomes. Researchers should employ standardized instruments such as the Research Impact Framework,^27^ which provide prompts and descriptive categories that would help them systematically identify a range of specific and verifiable implications related to their work (compared with *ad hoc* approaches that are often used). The framework could also help researchers think through implementation strategies and identify unintended or harmful effects.

The relatively small sample size (89 respondents out of about 400 Nigerian ophthalmologists) and the selection bias associated with obtaining information from only those who agreed to participate in a research study represent the limitations in this study.

In summary, study respondents indicated that the highest priority should be given to childhood blindness. The few that indicated some enthusiasm for research were largely involved in simple low-budget studies. They indicated that they are generally poorly published and tend to favor the local publication media. They also indicated that their research very rarely resulted in inventions (e.g., patented medication, instruments), change in clinical practice or health policy.

We recommend an (re)awakening of the enthusiasm of ophthalmologists in Nigeria for quality- and community-relevant research through retraining and emphasis on research training during both undergraduate and postgraduate medical education in Nigeria. Adequate resources and research infrastructures, particularly research funding, need to be provided for ophthalmologists in Nigeria.

## References

[1] Federal Republic of Nigeria: 2006 population census.

[2] Kyari F, Gudlavalleti MV, Sivsubramaniam S, Clare E, Gilbert CE, Abdull MM (2009). Nigeria National Blindness and Visual Impairment Study Group. Prevalence of Blindness and Visual Impairment in Nigeria: The National Blindness and Visual Impairment Survey. IOVS.

[3] Mahmoud AO, Kuranga SA, Ayanniyi AA, Babata AL, Adido J, Uyanne IA (2010). Appropriateness of ophthalmic cases presenting to a Nigerian tertiary health facility: Implications for service delivery in a developing country. Niger J Clin Pract.

[4] (2000). World Health Organization: WHO medicines strategy: Framework for action in essential drugs and medicine policy 2000-2003.

[5] (2005). World Health Organization: The World Health Report: Making every mother and child count.

[6] (2004). United Nations Development Programme: Human Development Report 2004: Cultural liberty in today’s diverse world.

[7] (2005). United Nations Development Programme: Human Development Report 2005: International cooperation at a crossroads: Aid, trade and security in an unequal world.

[8] (2006). Transparency International: Global corruption report 2006.

[9] Kirigia JM, Wambebe C (2006). Status of national health research systems in ten countries of the WHO African Region. BMC Health Serv Res.

[10] Uthman OA, Uthman MB (2007). Geography of Africa biomedical publications: An analysis of 1996–2005 Pub Med papers. Int J Health Geogr.

[11] Sabzwari S, Kauser S, Khuwaja AK (2009). Experiences, attitudes and barriers towards research amongst junior faculty of Pakistani medical universities. BMC Med Educ.

[12] Page J, Heller RF, Kinlay S, Lim LL, Qian W, Suping Z (2003). Attitudes of developing world physicians to where medical research is performed and reported. BMC Public Health.

[13] Shewan LG, Glatz JA, Bennett CC, Coats AJ (2005). Contemporary (post-Wills) survey of the views of Australian medical researchers: Importance of funding, infrastructure and motivators for a research career. Med J Aust.

[14] Kavallaris M, Meachem SJ, Hulett MD, West CM, Pitt RE, Chesters JJ (2008). Perceptions in health and medical research careers: The Australian Society for Medical Research Workforce Survey. Med J Aust.

[15] Zinner DE, Campbell EG (2009). Life-Science Research within US Academic Medical Centers. JAMA.

[16] Wong TY, Tan DT (2003). The SERI-ARVO Meeting and future challenges of ophthalmic research in Asia. Br J Ophthalmol.

[17] Stefansson E, Zetterstrom C, Ehlers N, Kiilgaard JF, la Cour M, Sigurdsson H (2003). Nordic research in ophthalmology. Acta Ophthalmol Scand.

[18] (2005). World Health Organization /International agency for the Prevention of Blindness. State of the World’s Sight 1999-2005.

[19] Sommer A Clinical Research- A Primer for Ophthalmologists. Prepared for the International Council of Ophthalmology.

[20] Hanney SR, González Block MA (2006). Building health research systems to achieve better health. Health Res Policy Syst.

[21] Bates I, Anson D, Bedu-Addo G, Agbenyega T, Osei-Akoto AW, Nsiah-Asare A (2007). Evaluation of a learner-designed course for teaching health research skills in Ghana. BMC Med Educ.

[22] Butler L (2002). A list of published papers is no measure of value. Nature.

[23] Butler L (2003). Academic reactions.Modifying publication practices in response to funding formulas. Res Eval.

[24] Butler L (2003). Explaining Australia’s increased share of ISI publications - the effects of funding formula based on publication counts. Res Policy.

[25] Misau YA, Al-Sadat N, Gerei AB (2010). Brain-drain and health care delivery in developing countries. J Public Health Afr.

[26] Kuruvilla S, Mays N, Pleasant A, Walt G (2006). Describing the impact of health research: A Research Impact Framework. BMC Health Serv Res.

[27] Arewa OP (2010). Bridging the gap between outputs of clinical research and utilization towards improved health care outcome in Nigerian hospitals. Niger J Clin Pract.

